# Risk Adjustment for Smoking Identified through Tobacco Use Diagnoses in Hospital Data: A Validation Study

**DOI:** 10.1371/journal.pone.0095029

**Published:** 2014-04-15

**Authors:** Alys Havard, Louisa R. Jorm, Sanja Lujic

**Affiliations:** Centre for Health Research, University of Western Sydney, Penrith, New South Wales, Australia; Kagoshima University Graduate School of Medical and Dental Sciences, Japan

## Abstract

Adjustment for the differing risk profiles of patients is essential to the use of administrative hospital data for epidemiological research. Smoking is an important factor to include in such adjustments, but the accuracy of the diagnostic codes denoting smoking in hospital records is unknown. The aims of this study were to measure the validity of current smoking and ever smoked status identified from diagnoses in hospital records using a range of algorithms, relative to self-reported smoking status; and to examine whether the misclassification of smoking identified through hospital data is differential or non-differential with respect to common exposures and outcomes. Data from the baseline questionnaire of the 45 and Up Study, completed by 267,153 residents of New South Wales (NSW), Australia, aged 45 years and older, were linked to the NSW Admitted Patient Data Collection. Patients who had been admitted to hospital for an overnight stay between 1 July 2005 and the date of completion of the questionnaire (1 January 2006 to 2 March 2009) were included. Smokers were identified by applying a range of algorithms to hospital admission histories, and compared against self-reported smoking in the questionnaire (‘gold standard’). Sensitivities for current smoking ranged from 59% to 84%, while specificities were 94% to 98%. Sensitivities for ever smoked ranged from 45% to 74% and specificities were 93% to 97%. For the majority of algorithms, sensitivities and/or specificities differed significantly according to principal diagnosis, number of comorbidities, socioeconomic status, residential remoteness, Indigenous status, 28 day readmission and 365 day mortality. The identification of smoking through diagnoses in hospital data results in differential misclassification. Risk adjustment based on smoking identified from these data will yield potentially misleading results. Systematic capture of information about smoking in hospital records using a mandatory item would increase the utility of administrative data for epidemiological research.

## Introduction

Using administrative health data for epidemiological studies presents a number of advantages. The whole of population coverage of these data collections allows for large sample sizes and eliminates the risk of selection bias which can arise from the requirement to seek individual consent. Studies can be conducted in a time- and cost-efficient manner because the data are already collected, and there is no potential for recall bias. As with all observational research, however, epidemiological studies based on administrative data are limited by the non-random allocation of participants to exposure groups. In order to minimise the impact of the potentially unequal distribution of confounding factors between groups, adjustments for the different risk profiles of patients should be made in analyses. Smoking is one of the most important factors to include in risk adjustment because it is so influential in terms of health [1]–[3], and its prevalence varies between groups within populations [4], [5]. With the exception of perinatal data collections, which capture information about smoking during pregnancy, Australian administrative hospital datasets do not contain specific items relating to smoking status. This has limited the capacity of studies using these data to adjust for the potentially confounding effect of smoking.

Some researchers have improvised by using the recording of smoking as a diagnosis in hospital records as an indicator of smoking status. In its simplest form, the presence of a smoking diagnosis in a patient’s most recent admission record has been taken as an indicator of smoking [6]. The other method involves linking multiple hospitalisation records belonging to the same patient and determining whether a smoking diagnosis is present on any of these records. This is referred to as using a lookback period [7], with lookback periods of 1 year [8] and 5 years [9]–[11] variously used to identify smoking.

Information about the quality of information derived from admission histories, using lookback methods, is becoming of increasing importance as more datasets are being linked together and as data linkage becomes more commonplace. The validity of identifying smoking from Australian hospital data using lookback methods has only been examined to some extent. Smoking diagnoses in any record since the inception of the Western Australian Hospital Morbidity Data Collection in 1979 (equating to lookback periods ranging from 16 to 24 years)were compared with self-reported history of ever smoking for a cohort of 12,203 men, yielding sensitivities of 26% to 48%, and specificities of 97% [12]. This information, however, is of limited utility as lookback periods of this length are not possible for the hospital data collections in many jurisdictions, for example, hospital data for New South Wales (NSW), Australia’s most populous State, are only available from July 2000. The validity of hospital records for ascertaining current smoking, which, in many studies, may be of greater relevance as a potential confounder than ever smoked status, has also not been measured.

What also remains to be investigated is whether the misclassification arising from less than perfect identification of smoking from hospital data is differential or non-differential. If the misclassification of a confounder is the same across all levels of the exposure and outcome (i.e. non-differential), the direction of the resulting bias is predictable. Specifically, there will be residual confounding such that the effect estimate falls between the unadjusted and adjusted effect [13]. Under these circumstances, there can still be value in adjusting for the misclassified confounder, as long as it is recognised that the effect estimate represents a partially adjusted effect. When the measurement error occurs to a different extent at different levels of the exposure or outcome, differential misclassification is present. This leads to biased allocation of participants into analysis strata, diluting or strengthening the association, or even producing a spurious one, such that the resulting effect estimate may not even fall between the unadjusted and adjusted effect [14]. Given the potential for adjustment for a differentially misclassified confounder to lead to erroneous conclusions, it is of utmost importance to establish whether the identification of smoking through hospital data results in differential misclassification of smoking.

This study aimed to address these gaps in knowledge regarding the validity of identifying smoking through algorithms based on smoking diagnoses in administrative hospital data. Specifically, the aims of this study were:

1. To measure the validity of both current smoking and ever smoked status identified from diagnoses in hospital records using the most recent separation as well as a range of lookback periods relative to self-reported smoking status; and
2. To examine whether the misclassification of smoking arising from the use of hospital data is differential or non-differential with respect to relevant outcomes and exposures.

## Methods

### Data Sources and Linkage

Baseline questionnaire data from the Sax Institute’s 45 and Up Study were linked to records from the NSW Admitted Patients Data Collection (APDC) and the NSW Register of Births Deaths and Marriages (RBDM).

The 45 and Up Study is a cohort study of men and women aged 45 and older and resident in NSW, Australia. Prospective participants were randomly sampled from the enrolment database of Medicare Australia, which provides near complete coverage of the population. People resident in non-urban areas and those aged 80 and older were oversampled. A total of 267,153 participants joined the Study by completing a baseline questionnaire (between January 2006 and December 2009) and giving signed consent for linkage of their information to routine health databases. About 18% of those invited participated, a response rate consistent with other cohort studies of this nature. Participants included about 10% of the NSW population aged 45 years and over. Baseline questionnaire data include information on key demographic and health-related factors, including Indigenous status, country of birth, household income, level of education, smoking, alcohol use, physical activity, height and weight and medical and surgical history. Further detail regarding the 45 and Up Study methods can be found elsewhere [15].

The APDC includes records for all hospital separations (discharges, transfers and deaths) from all NSW public and private sector hospitals and day procedure centres. The information reported includes patient demographics, source of referral to the service, service referred to on separation and diagnoses (up to 55), procedures (up to 50), and external causes of injury coded according to the Australian modification of the International Statistical Classification of Diseases and Related Problems, 10^th^ revision (ICD-10-AM) [16]. The APDC data used in the current study related to all separations between 1 July 2000 and 31 December 2010 (inclusive).

The NSW RBDM captures details of all deaths registered in NSW. The data used in the current study related to deaths of 45 and Up Study participants between 1 January 2006 and 16 June 2011.

Probabilistic linkage of these datasets was performed by the Centre for Health Record Linkage (CHeReL) using the ‘best practice’ protocol for preserving individual privacy [17]. Quality assurance data show false positive and negative rates for data linkage of 0.4% and less than 0.1%, respectively.

### Sample

45 and Up Study participants who had been admitted to a NSW hospital for an overnight stay at least once between July 1 2005 and the date of completion of the 45 and Up Study questionnaire were eligible for inclusion in this validation study. An admission after 1 July 2005 was an eligibility criterion to ensure that 5 years of hospitalisation lookback was available for all participants. After excluding 3173 people because responses to the smoking items in the questionnaire data were incomplete or inconsistent with each other, the final sample comprised 63,355 participants.

### Measures

Self-reported smoking in the 45 and Up Study baseline questionnaire was used as the gold standard. For each participant, the most recent hospital separation was identified as their hospital record with a separation date prior to, but as close as possible to, their survey completion date. The age of the participant at the time of this separation was extracted. The questions “have you ever been a regular smoker?” and “are you a regular smoker now?” were then used in combination with “how old were you when you started smoking regularly?” and “how old were you when you stopped smoking?” to determine whether participants had ever been a regular smoker at the time of their most recent hospital separation (ever smoked) and whether they were regular smokers at the time of their most recent separation (current smoking) (further details and an illustration provided in Figure S1). Participants were excluded from the current smoking analyses if the age s/he reported starting (n = 2) or stopping (n = 271) smoking was the same as their age at their most recent hospitalisation. The two participants who started smoking at the same age as their most recent hospitalisation were excluded from the ever smoked analyses.

Four algorithms (presented in Table 1) for classifying an individual as a smoker or non-smoker based on hospital data were applied to participants’ records of separations between 1 July 2000 and the date the participant completed the survey. These 424,836 APDC records related to 395,908 hospitalisation episodes after grouping admissions relating to a single hospital stay (where multiple records arose due to transfers between hospitals).

**Table 1:** Algorithms for identifying smokers from hospital data.

Algorithm	Identified as a smoker if:
Most recent separation	Smoking diagnosis was present in hospital record with a separation date prior to, but as close as possible to, the survey completion date
Most recent episode	Smoking diagnosis was present in any of the record(s) comprising the episode with a summary separation date prior to, but as close as possible to, the survey completion date
1 year lookback	Smoking diagnosis was present in the most recent episode or in at least one hospital record with a separation date in the 365 days prior to the separation date of the most recent episode
5 year lookback	Smoking diagnosis was present in the most recent episode or in at least one hospital record with a separation date in the 5 years (1826 days) prior to the separation date of the most recent episode.

An ever smoked diagnosis was considered present in a hospital record when an ICD-10-AM code of F17.1, F17.2, Z72.0 or Z86.43 was recorded in any diagnosis field (see Table 2 for a description of these diagnosis codes). A current smoking diagnosis was considered present when an ICD-10-AM code of F17.2 or Z72.0 was present in any diagnosis field.

**Table 2:** ICD-10-AM diagnosis codes relating to smoking.

ICD-10-AM code	Description
F17.1	Harmful use of tobacco. Assigned if the clinician has clearly documented a relationship between a particular condition(s) and smoking – even if the patient has ceased smoking
F17.2	Tobacco dependence syndrome
Z72.0	Tobacco use, current. Assigned if the patient has smoked any amount of tobacco within the last month
Z86.43	Personal history of tobacco use disorder. Assigned if it is documented that the patient smoked any amount of tobacco in the past, but excluding the last month

### Assessment of Validity

The extent to which each algorithm correctly identified smoking status was assessed using sensitivity, specificity and positive predictive value (PPV), with 95% confidence intervals, and the kappa statistic. *Sensitivity* refers to the percentage of smokers (according to self-report) who were correctly identified as smokers by the algorithm. *Specificity* is the percentage of non-smokers (according to self-report) who were correctly identified as non-smokers by the algorithm. *PPV* is the percentage of those identified as smokers by the algorithm who were indeed smokers (according to self-report). The kappa statistic (κ) is the chance-corrected proportional agreement. A κ value of 0.75 or higher indicates excellent agreement beyond chance, a κ between 0.4 and 0.75 indicates good agreement and a κ less than 0.4 indicates poor agreement [18].

### Characterising Misclassification as Differential or Non-differential

In order to assess whether the application of the algorithms resulted in differential misclassification of smoking, sensitivity and specificity calculations were stratified for the commonly used outcomes of 28 day readmission and 365 day mortality, both as binary measures. Participants with at least one APDC record where the admission date was within 28 days of the separation date associated with their most recent hospitalisation episode (as defined in Table 1) were identified as having a 28 day readmission. Similarly, 365 day mortality was measured through RBDM records where the date of death was within 365 days of the separation date associated with the most recent hospitalisation episode. Calculations were also stratified by exposures, selected on the basis of commonly studied exposure-outcome relationships which might be confounded by smoking. These included socioeconomic status (SES), remoteness of residence, Indigenous status, principal diagnosis and number of comorbidities. Information regarding each of these exposures was obtained from the participant’s most recent separation record. SES was classified according to the Socioeconomic Indexes for Areas (SEIFA), Index of Relative Socioeconomic Advantage [19] mapped to statistical local area (SLA) of residence and grouped into quintiles. Remoteness of residence was classified according to the Accessibility and Remoteness Index of Australia (ARIA) [20] applied to SLA of residence, and grouped into four categories (major city, inner regional, outer regional, and remote/very remote). Principal diagnoses were categorised into 19 ICD-10-AM chapter headings. The number of Charlson Index comorbidities [21], ascertained from additional diagnoses, was categorised as none, one, two and three or more. For each exposure and outcome examined, an algorithm was identified as resulting in differential misclassification when its sensitivity and/or specificity differed between at least two levels of the exposure or outcome variables, as indicated by non-overlapping 95% confidence intervals [22]. As PPV is a function of the prevalence of the behaviour being measured, and the prevalence of smoking is likely to differ between levels of the exposure and outcome variables compared, PPV was not considered an appropriate measure for characterising misclassification.

In order to assess whether the finding of differential or non-differential misclassification might be accounted for by the unequal distribution of factors related to the accuracy of diagnostic coding [23], [24], sensitivity and specificity calculations were further stratified by the diagnosis type (surgical, medical or other) and the hospital type (public or private) of the most recent admission.

All analyses were conducted in SAS, version 9.3 [25]. The conduct of the 45 and Up Study was approved by the University of New South Wales Human Research Ethics Committee (HREC), while ethical approval for this particular study was provided by the NSW Population and Health Services Research Ethics Committee and the University of Western Sydney HREC. Written informed consent was given by participants for their health records to be used in this study, and data were de-identified prior to release to the researchers. The dataset was constructed with the permission of each of the data custodians of the respective source datasets and with specific ethical approval. The dataset could potentially be made available to other researchers if they obtain the necessary approvals. More information about these approvals is available from the authors on request.

## Results

### Sample Characteristics

The sample comprised 63,355 participants, with a median of 4 hospitalisation episodes per person between 1 July 2000 and the date the participant completed the survey. The characteristics of the sample, at the time of their most recent hospitalisation, are presented in Table 3. The mean age of participants was 66 years, 49% were male, 6% were regular smokers at the time of the survey and 44% reported ever being a regular smoker.

**Table 3:** Characteristics of participants at the time of their most recent hospital admission.

	n	%
**Sex**		
Male	31,302	49
Female	32.053	51
**Age**		
≤54	13,711	49
55–64	17,396	51
65–74	15,773	25
75–84	13,451	21
85+	3,024	5
**Socioeconomic status**		
Decile 1–2	6,658	11
Decile 2–4	10,962	17
Decile 5–6	14,812	23
Decile 7–8	11,474	18
Decile 9–10	16,514	26
Missing	2,935	5
**Indigenous status**		
Aboriginal or Torres Strait Islander	268	<1
Not Aboriginal or Torres Strait Islander	62,285	98
Missing	802	1
**Remoteness of residence**		
Major city	26,979	43
Inner regional	24,341	38
Outer regional	11,058	17
Remote/very remote	673	1
Missing	304	<1
**Smoking (at the time of survey completion)**		
Currently a regular smoker	3,852	6
Ever been a regular smoker	27,905	44
**Principal diagnosis**		
Infectious & parasitic	598	1
Neoplasms	5,684	9
Blood & immune mechanism	502	1
Endocrine, nutritional & metabolic	1,302	2
Mental & behavioural	972	2
Nervous system	2,051	3
Eye & adnexa	2,289	4
Ear & mastoid process	440	1
Circulatory	7,580	12
Respiratory	2,893	5
Digestive	9,408	15
Skin & subcutaneous	1,055	2
Musculoskeletal	6,964	11
Genitourinary	4,932	8
Pregnancy & childbirth	56	<1
Congenital malformations	86	<1
Symptoms & findings NEC	5,447	9
Injury & poisoning	4,553	7
Factors influencing health care	6,503	10
Missing	40	<1
**Number of comorbidities**		
0	56,255	89
1	4,017	6
2	1,761	3
3+	1,322	2
**28 day readmission**		
No	62,547	99
Yes	787	1
Died	21	<1
**365 day mortality**		
No	62,281	99
Yes	674	1

### Validity of Smoking Status

Table 4 summarises the validation statistics for the ascertainment of smoking, separately for current smoking and ever smoked. According to the kappa statistic, all algorithms had good agreement beyond chance with self-reported smoking status. Sensitivities for the ascertainment of current smoking produced by the four algorithms ranged from 59% to 84%, with the lowest sensitivities found when current smoking diagnoses were retrieved from the most recent separation or hospitalisation episode record. Sensitivity increased significantly with longer lookback periods. Specificities for the ascertainment of current smoking ranged from 94% to 98% and PPVs ranged from 51% to 72%, with both significantly decreasing as the lookback period was extended.

**Table 4:** Performance of algorithms for identifying current smoking and ever smoked status from APDC records.

	Algorithm	N	+ve45 Up	+veAPDC	+veboth	Sn %(95% CI)	Sp %(95% CI)	PPV %(95% CI)	Kappa
**Current smoking**	**Most recent separation**	63082	4223	3425	2471	58.5 (56.9–60.2)	98.4 (98.3–98.5)	72.2 (70.8–73.5)	0.62
	**Most recent episode**			3545	2533	60.0 (58.4–61.6)	98.3 (98.2–98.4)	71.5 (70.1–72.8)	0.63
	**1 yr lookback**			4745	3045	72.1 (70.8–73.4)	97.1 (97.0–97.2)	64.2 (62.7–65.6)	0.65
	**5 yr lookback**			6925	3531	83.6 (82.7–84.5)	94.2 (94.0–94.4)	51.0 (49.5–52.5)	0.60
**Ever smoked**	**Most recent separation**	63353	27899	13549	12468	44.7 (43.9–45.5)	97.0 (96.8–97.1)	92.0 (91.7–92.3)	0.44
	**Most recent episode**			14085	12939	46.4 (45.6–47.2)	96.8 (96.6–97.0)	91.9 (91.5–92.2)	0.46
	**1 yr lookback**			18569	16802	60.2 (59.5–60.9)	95.0 (94.8–95.2)	90.5 (90.1–90.8)	0.57
	**5 yr lookback**			23307	20644	74.0 (73.4–74.6)	92.5 (92.2–92.8)	88.6 (88.2–88.9)	0.68

Sensitivities for the ascertainment of ever smoked ranged from 45% to 74%. Sensitivities increased significantly as more records were searched. Specificities for the ascertainment of ever smoked ranged from 93% to 97% and PPVs ranged from 87% to 92%. Again, both specificity and PPV decreased significantly as the lookback period was extended.

### Characterisation of Misclassification

The sensitivity and/or specificity of the algorithms differed significantly between at least two levels of most of the exposures and outcomes examined (Table 5). In addition to the large amount of differential misclassification identified through non overlapping 95% confidence intervals, it should be noted that in some instances where misclassification was not characterised as differential, point estimates differed substantially but 95% confidence intervals were wide and overlapped due to a limited number of participants in that particular stratum (detailed data presented in Table S1).

**Table 5:** Whether the misclassification of smoking arising from application of the algorithms is differential between levels of common exposures and outcomes.

		SES	Residential remoteness	Indigenous status	Diagnosis	Number of comorbidities	28 day readmission	365 day mortality
**Current smoking**	**Most recent separation**	✓		✓	✓	✓	✓	
	**Most recent episode**	✓		✓	✓	✓	✓	
	**1 yr lookback**	✓	✓	✓	✓	✓	✓	
	**5 yr lookback**	✓		✓	✓	✓	✓	✓
**Ever smoked**	**Most recent separation**	✓	✓	✓	✓	✓	✓	
	**Most recent episode**	✓	✓	✓	✓	✓	✓	
	**1 yr lookback**	✓	✓	✓	✓	✓		✓
	**5 yr lookback**	✓	✓	✓	✓	✓	✓	✓

✓indicates the presence of differential misclassification, defined as a difference in sensitivity and/or specificity between at least two levels on the exposure/outcome, as indicated by non-overlapping 95% confidence intervals.

General patterns included current smoking being identified with higher specificity among patients in the highest SES stratum relative to the lowest, and ever smoked being identified with lower sensitivity among patients in the second lowest SES stratum relative to the higher SES strata. Ever smoked was identified with higher sensitivity in residents of major cities relative to regional and remote areas, as was ever smoked in inner regional areas relative to outer regional and remote areas. The specificity of ever smoked was lower among residents of major cities relative to outer regional areas, and for certain algorithms, lower in residents of major cities relative to inner regional areas. The sensitivity of current smoking was generally higher in Indigenous patients relative to those for whom Indigenous status was missing, while no clear pattern emerged for current smoking among Indigenous participants. The sensitivity of ever smoked was generally higher in both Indigenous and non-Indigenous participants relative to those for whom Indigenous status was missing. Both current smoking and ever smoked were identified with higher specificity among non-Indigenous participants relative to Indigenous participants. The algorithms based on the most recent separation and most recent episode identified current smoking with higher sensitivity for patients with neoplasms and diseases of the circulatory or digestive system relative to patients with diseases of the nervous system. Similarly, these algorithms identified ever smoked with higher sensitivity among patients with neoplasms and diseases of the circulatory, digestive or musculoskeletal system relative to patients with all other diagnoses. These algorithms identified current smoking with lower specificity among patients with mental and behavioural disorders and diseases of the circulatory or respiratory system relative to patients with other diagnoses. The specificity of ever smoked was lower among patients with neoplasms and diseases of the circulatory, digestive or musculoskeletal system compared with other diagnoses. There appeared to be no consistent pattern according to diagnosis for the sensitivities of the algorithms including a lookback period, while these algorithms identified current smoking with lower specificity among patients with mental and behavioural disorders, and ever smoked with lower specificity among patients with diseases of the circulatory system relative to patients with most other diagnoses. No consistent pattern emerged in the sensitivity of identifying current smoking according to the number of comorbidities, while ever smoked was identified with higher sensitivity among patients with one recorded comorbidity than among patients without any comorbidities. The specificity of identifying both current smoking and ever smoked was lower among patients with one recorded comorbidity than among patients without any comorbidities. The sensitivity of identifying current smoking was higher for patients who were readmitted within 28 days, relative to those who were not. No consistent pattern emerged with regards to the sensitivity of identifying ever smoked according to readmission status, nor the specificity of identifying either current smoking or ever smoked. There was no consistent pattern in the sensitivities or specificities of identifying current smoking or ever smoked according to 365 day mortality.

When sensitivity and specificity calculations were further stratified by diagnosis type and hospital type, significant differences between at least two levels of most exposures and outcomes remained. For the majority of instances where significant differences were no longer detected, stratum-specific sample sizes were small and substantial disparities in the point estimates were observed (see detailed data in Table S1).

## Discussion

This study showed that the validity of smoking status ascertained through hospital data varies according to the algorithm applied. Restricting the search for smoking diagnoses to the records relating to the most recent separation or hospitalisation episode yielded the lowest sensitivities, and the highest specificities and PPVs, while algorithms in which all records in a lookback period were searched had the highest sensitivities and the lowest specificities and PPVs. For all algorithms, current smoking was ascertained with higher sensitivity and specificity than ever smoked.

To provide an indication of the extent of risk adjustment that is achievable with smoking ascertained through diagnoses in hospital data, our sensitivity and specificity estimates can be plotted against those from a published scenario analysis. The scenario is one in which smoking is a confounder in the relationship between coffee consumption and bladder cancer, and the amount of confounding bias removed at different levels of smoking misclassification is quantified [26]. For both current smoking and ever smoked, the 5 year lookback algorithm would result in less residual confounding than the other algorithms. Specifically, for the scenario presented, approximately 35% of the original bias associated with current smoking would remain after adjusting for smoking ascertained with the 5 year lookback algorithm. Approximately 50% of the bias associated with ever smoked would remain if the adjustment was conducted with ever smoked ascertained using the 5 year lookback algorithm. Although these figures represent estimates of residual confounding in a particular set of circumstances, the authors markedly varied all parameters in the scenario and found little fluctuation in the extent of risk adjustment achieved at each level of smoking misclassification [26].

Although the validation statistics from the first aim of this study suggest there could be value in adjusting for smoking ascertained through hospital data, to achieve at least partial adjustment for this key confounder, the findings from the second aim of the study indicate this is not the case. The generalisation that an imperfectly classified confounder results in residual confounding only holds if the misclassification is the same across all levels of the exposure and outcome [13]. In the current study, the sensitivity and/or specificity of algorithm-identified current smoking and ever smoked differed significantly between at least two levels of many common exposures and outcomes. Further stratification of the validation statistics indicated that the presence of differential misclassification was not due to an unequal distribution of diagnosis types and hospital types across levels of the exposures and outcomes. Given that adjustment for a differentially misclassified confounder can lead to unpredictable biases and spurious results, risk adjustment for smoking identified through diagnoses in hospital data is not recommended without some quantitative analysis of potential biases. Techniques exist for estimating the magnitude and direction of potential biases introduced by a differentially misclassified confounder, based on sensitivity and specificity estimates from an internal validation sub-study or a range of plausible values derived from an external validation study such as this (see e.g. [14]).

Of course, it always preferable to eliminate measurement error rather than using statistical fixes. This would require improved recording of patients’ smoking status in administrative hospital data uniformly across all patient groups. It is not realistic to expect that this be achieved by requiring systematic recording of smoking diagnoses in hospital records for all current and past smokers because of the Australian coding standard dictating that additional diagnoses only be assigned if they affect patient care during that admission [16]. Instead, we propose that administrative hospital databases be expanded to include a mandatory data item regarding the patients’ smoking status (current, former, never smoker) at the time of admission. This would require systematic collection and recording of smoking information by admissions or clinical staff at some stage during a patient’s hospital stay, followed by transfer of this information to administrative hospital databases. A study of 169 publicly funded hospitals in NSW indicates that this information is already collected to some extent, with 80% of senior hospital managers reporting that smoking status is recorded for 80–100% of their patients [27]. The data collection methods currently being used may need to be revised to improve the accuracy of this information, as data collected by admissions clerks in a single Australian hospital identified only 63% of current smokers, while reporting to treating doctors was much more accurate [28]. Impetus to improve the coverage and accuracy of this information collection is provided not only by the plethora of epidemiological studies based on hospital data, but by clinical care guidelines, which recommend that every tobacco user be identified on admission to promote appropriate management of potential nicotine dependence [29], [30]. Moreover, the advantages to public health research arising from systematically recorded smoking status on hospital records are not limited to the ability to adjust for the potentially confounding effect of smoking. It would also allow for the direct measurement of the burden associated with tobacco use in Australia, in a context where the current reliance on indirect estimation is recognised to be problematic [31].

There are potential limits to the generalisability of this study’s findings, as analyses were based on participants in the 45 and Up Study. Two-thirds of hospital separations in Australia are for patients 45 years and older [32], with the prevalence of smoking lower for people in this age group [33]. Furthermore, the 45 and Up Study had a response rate of 18%, similar to other cohort studies of this nature. Differences in the prevalence of regular smoking between 45 and Up Study participants (weighted for age, sex and remoteness) and respondents of the most comparable population survey (7.5% vs 12%) [34], indicate that the study participants are not representative of the general population in terms of smoking behaviour. Whether the selectiveness of this group impacts on rates of agreement between self-reported smoking status and hospital recorded smoking diagnoses, however, is not clear.

Consideration should also be given to the extent to which the findings are generalisable to administrative data from other Australian States and Territories, and from other countries. The allocation of diagnostic codes is likely to differ according to the coding systems and standards in place, whether the person responsible for assigning a code is a trained professional coder, the strength and scope of incentives for coding, and the number of diagnosis fields available [35]. Consistency between data collections within Australia is probable given the ICD-10-AM and national coding standards are applied by trained coders nationwide, the funding models in all States and Territories are, at least in part, activity-based [36], and all databases have at least 20 diagnosis fields, where a mean of three diagnoses are allocated per separation [37].

An additional limitation arises from the use of a self-reported measure of smoking as a gold standard, which has a mean sensitivity and specificity of 88% and 89% when measured against biochemical assessment [38]. For participants who had changed their smoking status in the 0.5–4 years between their most recent hospital admission and completing the survey (n = 381, 0.6%), his/her current smoking status at the time of admission was extrapolated from the age he/she reported starting and stopping smoking. As retrospectively collected smoking status shows only moderate agreement with data collected prospectively [39], the accuracy of the gold standard may have been lower for these participants.

Finally, the small sample sizes in certain strata when calculations were stratified by exposures and outcomes, and then further stratified by diagnosis type and hospital type, yielded imprecise estimates of sensitivity and specificity (as indicated by wide confidence intervals). If the strata with small sample sizes were removed from consideration, however, much non-overlap of confidence intervals would still have been observed, and the same conclusion regarding the presence of differential misclassification for most exposures and outcomes would still have been drawn.

Despite these limitations, this study presents data that are an improvement on currently available information regarding the quality of smoking information in hospital records. Prior evaluations include the measurement of agreement between smoking diagnoses in administrative hospital data and auditor allocated diagnoses [40], and a single study validating hospital recorded smoking against an independent source, but this was based on a smaller sample (N = 12,203) comprising only men aged 65 to 83 years [12]. Other studies have not focused specifically on administrative hospital data, instead examining the validity of smoking diagnoses in administrative clinic and Veterans Health Administration data [41], [42]. As the only study to examine a range of algorithms for identifying smoking from administrative hospital data, including an assessment of whether the misclassification produced by these measures is differential, our study has provided new evidence that should be taken into account in future studies based on administrative hospital data. It suggests that risk adjustment based on smoking identified from diagnoses in these data will yield potentially misleading results, and echoes recommendations made in clinical guidelines regarding the need to systematically identify and record smoking status in hospital records.

## Supporting Information

Figure S1 **The gold standard definition of ever smoked and current smoking status.**
(DOCX)

Table S1 **Sensitivities and specificities of the algorithms, stratified by socioeconomic status (SES), remoteness of residence, Indigenous status, principal diagnosis, number of comorbidities, 28 day readmission, 365 day mortality, and further stratified by diagnosis type and hospital type.**
(XLSX)
