# Supplementary material for: Risk Adjustment for Smoking Identified through Tobacco Use Diagnoses in Hospital Data: A Validation Study
Source: PLoS One. 2014 Apr 15;9(4):e95029. doi: 10.1371/journal.pone.0095029 (PMC3988140; doi:10.1371/journal.pone.0095029)

**Figure S1: The gold standard definition of ever smoked and current smoking status**

As depicted in Figure S1, all participants who responded yes to the first question were classified as ever smokers (scenario 1), unless the age at which s/he reported started smoking was older than the age at which s/he was most recently hospitalised (scenario 2). Participants who responded yes to the first and third question were classified as current smokers (scenario 1), unless the age at which s/he reported started smoking was older than the age at which s/he was most recently hospitalised (scenario 2). Participants who responded yes to the first question and no to the third question were also classified as current smokers if the age when s/he stopped smoking was older than the age at which s/he was most recently hospitalised (scenario 3). If the age at which s/he stopped was younger than the age at which s/he was hospitalised, the participant was not classified as a current smoker (scenario 4). Participants were excluded from the current smoking analyses if the age s/he reported starting (n = 2) or stopping (n = 271) smoking was the same as their age at their most recent hospitalisation (scenario 5 and 6 respectively). The two participants who started smoking at the same age as their most recent hospitalisation were excluded from the ever smoked analyses (scenario 5).


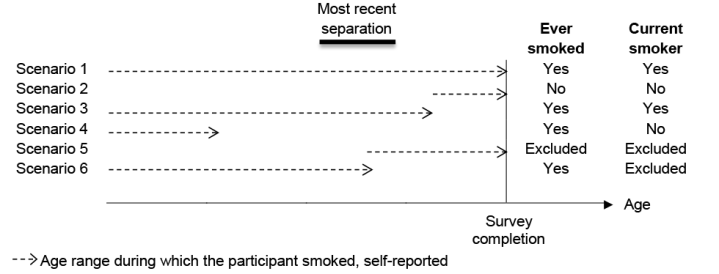

Supplement: Figure S1 — The gold standard definition of ever smoked and current smoking status. (DOCX) [file pone.0095029.s001.docx]
